# A Case Study for Large-Scale Human Microbiome Analysis Using JCVI’s Metagenomics Reports (METAREP)

**DOI:** 10.1371/journal.pone.0029044

**Published:** 2012-06-13

**Authors:** Johannes Goll, Mathangi Thiagarajan, Sahar Abubucker, Curtis Huttenhower, Shibu Yooseph, Barbara A. Methé

**Affiliations:** 1 The J. Craig Venter Institute, Rockville, Maryland, United States of America; 2 The J. Craig Venter Institute, San Diego, California, United States of America; 3 The Genome Institute, Washington University School of Medicine, St. Louis, Missouri, United States of America; 4 Harvard School of Public Health, Boston, Massachusetts, United States of America; Emory University School of Medicine, United States of America

## Abstract

As metagenomic studies continue to increase in their number, sequence volume and complexity, the scalability of biological analysis frameworks has become a rate-limiting factor to meaningful data interpretation. To address this issue, we have developed JCVI Metagenomics Reports (METAREP) as an open source tool to query, browse, and compare extremely large volumes of metagenomic annotations. Here we present improvements to this software including the implementation of a dynamic weighting of taxonomic and functional annotation, support for distributed searches, advanced clustering routines, and integration of additional annotation input formats. The utility of these improvements to data interpretation are demonstrated through the application of multiple comparative analysis strategies to shotgun metagenomic data produced by the National Institutes of Health Roadmap for Biomedical Research Human Microbiome Project (HMP) (http://nihroadmap.nih.gov). Specifically, the scalability of the dynamic weighting feature is evaluated and established by its application to the analysis of over 400 million weighted gene annotations derived from 14 billion short reads as predicted by the HMP Unified Metabolic Analysis Network (HUMAnN) pipeline. Further, the capacity of METAREP to facilitate the identification and simultaneous comparison of taxonomic and functional annotations including biological pathway and individual enzyme abundances from hundreds of community samples is demonstrated by providing scenarios that describe how these data can be mined to answer biological questions related to the human microbiome. These strategies provide users with a reference of how to conduct similar large-scale metagenomic analyses using METAREP with their own sequence data, while in this study they reveal insights into the nature and extent of variation in taxonomic and functional profiles across body habitats and individuals. Over one thousand HMP WGS datasets and the latest open source code are available at http://www.jcvi.org/hmp-metarep.

## Introduction

Several large scale metagenomic studies have been completed or are underway to investigate the genetic composition of microbes in their natural environment. Prominent efforts include the Global Ocean Sampling [1]–[3], interrogations of a variety of diverse environments [4]–[6] and more recently the human microbiome [7], [8]. Increasingly such work is planned and carried out as part of larger consortia and funding efforts. Examples include MetaHIT [7], the Earth Microbiome Project [9], http://www.terragenome.org, and the HMP [10]. The HMP, represents an effort to characterize the microbial communities associated with multiple habitats across the human body, and is an excellent example of the complexity, scale and nature of such projects and consortia. With its focus on the resident bacteria of so called normal donors, this project provides a critical baseline for future metagenomic studies of the human microbiome including their associations with human health and disease. As a multi-faceted community resource, the HMP includes taxonomic marker studies of 16S rRNA gene sequences [11] as well as a whole genome shotgun (WGS) data survey [10], [12]–[15]. This WGS metagenomic data survey has examined the taxonomy and functional potential of microbial communities from 741 samples taken from up to fifteen body habitats of 108 healthy adult men and women generating in total approximately 38 billion short read sequences (3.5 Tbp) of which over 14 billion sequences were processed and analyzed as a part of this study. This information is complementary to 16S rRNA gene based organismal identifications and other taxonomic marker sequences, however the task of annotating and characterizing large collections of such data is similarly challenging.

To identify taxonomic and functional signatures, WGS metagenomic data are curated by either directly annotating short reads [16], [17] or, as would be performed for the sequenced genome of a single organism, annotated post assembly taking advantage of the larger contigs [18]. Annotation of these data is a computationally intensive activity, which requires extensive BLAST-like homology searches that can be difficult both to perform and store. Fortunately, billions of short sequence reads can be most usefully analyzed after condensing the data to taxonomic, enzymatic, and/or pathway abundances, which can subsequently be studied more efficiently.

To provide a computational framework within which to perform such tasks, we have developed JCVI Metagenomics Reports (METAREP), an open source tool for high-performance comparative metagenomics [19]. The software utilizes a scalable data warehouse solution that allows effective storage and dynamic querying of annotation data that can be produced by various annotation methods. The data model of METAREP version 1.3.1, presented in this report, has been expanded to allow the direct importation and analysis of results produced by two annotation pipelines used in the HMP: (1) JCVI’s Prokaryotic Metagenomics Annotation Pipeline (JPMAP) [18] used for the annotation of open reading frames from assemblies and (2) HUMAnN [16] to annotate short reads. In addition, frequencies of functional and taxonomic attributes can be adjusted using custom annotation weights. The scalability of such weighted frequency calculations has been improved by utilizing distributed searches.

In this study, we present advancements to the METAREP software focusing on the implementation of an extended data model, improved scalability and analytical features which have facilitated biological comparisons and interpretation of human microbiome metagenomic data generated by the HMP across multiple samples, body habitats and individuals. In particular, we introduce several biological scenarios and hypotheses along with appropriate analytical strategies designed to investigate these questions as well as demonstrate important downstream, analytical features of METAREP including: how to filter the data for enzymatic markers, visualize marker composition across organisms and human habitats, conduct hierarchical clustering analysis of individual samples, and carry out non-parametric statistical analyses to detect differentially abundant taxa and pathways in oral habitats. The results of these scenarios provide templates of analytical strategies for future users of METAREP that can be applied to similar data. Further, the results of the current scenarios have revealed new insights into the taxonomic and functional relationships between multiple body habitats and individuals of the human microbiome. Finally, we also provide specific descriptions of software architecture improvements and results of tests designed to benchmark performance response time of the software. Overall, this work introduces an important software tool and strategies for comparative analysis of large-scale metagenomic data generated from complex experimental designs.

## Results

### Human Microbiome Case Study

For this case study, we have established a dedicated instance of our software (version 1.3.1) to host HMP WGS annotations at http://www.jcvi.org/hmp-metarep. The HMP METAREP instance currently allows interactive data analysis of over 400 million weighted gene annotations predicted from 14 billion short-reads by HUMAnN as well as ORF-based annotations predicted from over 700 assemblies by JPMAP (Table 1). Each annotation entry may possess multiple attributes. Supported attributes range from organismal information (NCBI taxonomy), to functional description, Enzyme Classification (EC), Gene Ontology (GO) [20] or KEGG Orthology (KO) [21] as well as KEGG and MetaCyc [22] pathway assignments. In addition, each annotation may be given a weight to adjust its overall abundance (see Methods section for dynamic weighting algorithm). Although outside the scope of the software, for completeness a brief description of the HMP WGS sequence generation, preprocessing, and annotation is summarized in the Methods section. After successful installation of the software, annotations can be imported and analyzed.

**Table 1:** Summary of available datasets by body habitat sorted by the number of WGS reads.

Habitat	#HUMANnN	#Reads	#Weighted Annotations	Sum of Annotation Weights	#Assembly
	Datasets	[million]	[million]	[million]	Datasets
Stool	68	6262	78	1563.8	151
Supragingival plaque	89	4192	112	1538.0	118
Buccal mucosa	116	1449	104	731.8	107
Tongue dorsum	23	1182	28	501.8	129
Right retroauricular crease	17	412	13.0	168.8	17
Posterior fornix	55	297	15	110.0	53
Anterior nares	91	164	38	38.5	87
Subgingival plaque	7	150	9	48.6	7
Left retroauricular crease	8	145	6	63.7	9
Palatine tonsils	6	135	6	54.7	6
Throat	6	129	6	53.1	7
Keratinized gingiva	2	45	2	23.9	6
Saliva	5	43	5	16.8	3
Vaginal introitus	3	6	1	1.8	3
Mid vagina	2	2	1	0.7	2
Total	498	14613	424	4916.0	705

Columns 2–5 refer to the HUMAnN datasets.

**Figure 1:**
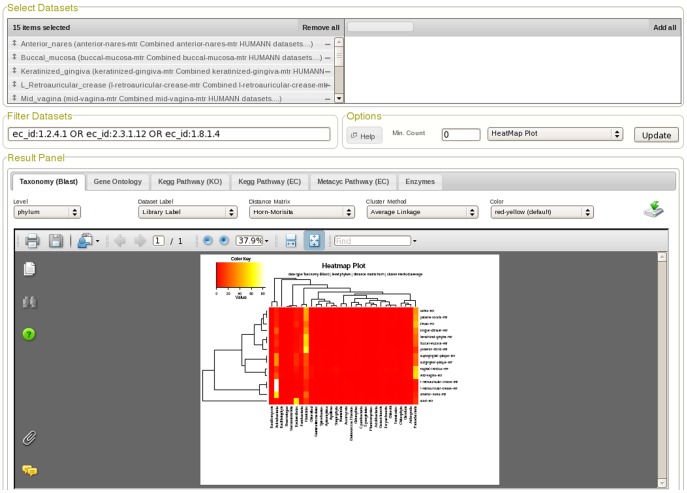
Screenshot of the METAREP Compare Page. The Compare page allows users to filter, compare and visualize annotation attributes across multiple datasets. As illustrated in the upper panel, the user can find and select datasets of interest (here pooled body habitats were selected). The middle panel illustrates filter and compare options (here datasets were filtered for the pyruvate dehydrogenase complex and the heatmap plot option was selected). The bottom panel shows the compare results and allows users to switch between annotation attributes and specify its level of granularity (here the taxonomy attribute and phylum level were selected).

In this report, we focus on analytical functions available through the METAREP Compare page which allows users to filter and compare multiple datasets and visualize differences using advanced visualization tools (Figure 1). Compare options include the generation of absolute and relative count summaries, hierarchical clustering, heatmaps, and multi-dimensional scaling plots, as well as the execution of statistical tests. Plots can be exported as publication ready PDF files while counts, distances matrices, and statistical results can be exported as text files. In the following, we describe three biological scenarios to highlight how these compare functions can be used for exploratory analysis of the weighted HUMAnN read based annotations.

### Scenario 1: Enzymatic Markers Contrasted Across Body Habitats and Taxa

#### Scenario 1 Introduction

Pyruvate is a key organic carbon intermediate centrally positioned at the intersection of assimilatory and dissimilatory pathways, and respiratory and fermentative metabolism [23]. As such, it can be expected to be important in the metabolism of the human microbiome. However, the specific enzymatic processes used for its metabolism and taxonomic membership are likely to vary across body habitats. To evaluate this hypothesis, three major enzymes of pyruvate metabolism: 1) pyruvate dehydrogenase complex (PDHC) [24] 2) pyruvate:ferredoxin oxidoreductase (PFOR) [25] and 3) pyruvate formate lyase (PFL) [26] have been examined for their relative abundance by taxonomic profiles and compared across multiple body habitats.

A common route of pyruvate metabolism is oxidative decarboxylation catalyzed by PDHC to yield the central intermediate acetyl-coenzyme A (CoA), which can be further oxidized through the TCA cycle, or used in anabolic pathways for synthesis of essential cell components, or carbon and energy storage compounds. The PDHC belongs to the family of 2-oxoacid dehydrogenase which consists of multi-subunit complexes responsible for the irreversible conversion of 2-oxoacids to their corresponding acyl-CoA derivatives. The PDHC is composed of three subunits, component E1, pyruvate dehydrogenase (1.2.4.1), component E2, dihydrolipoyl transacetylase (2.3.1.12) and component E3, dihydrolipoyl dehydrogenase (1.8.1.4) [27]. A key enzymatic counterpart to the PDHC in energy metabolism under anaerobic conditions is PFOR (1.2.7.1) which catalyzes a reversible, CoA-dependent oxidative decarboxylation of pyruvate yielding acetyl-CoA and CO_2_. As a reversible reaction, this enzyme also mediates the main CO_2_ fixing reaction for methanogens and a variety of photosynthetic organisms [28]. In contrast, some bacteria are capable of fermentation in which organic intermediates of metabolism such as pyruvate, serve as electron acceptors in the maintenance of overall redox balance; while ATP needed for cell growth is derived from substrate-level phosphorylation. PFL (2.3.1.54), a homodimer, catalyzes the reversible reaction of pyruvate and CoA into acetyl-CoA and formate [29].

#### Scenario 1 METAREP Analytical Methods

**Figure 2:**
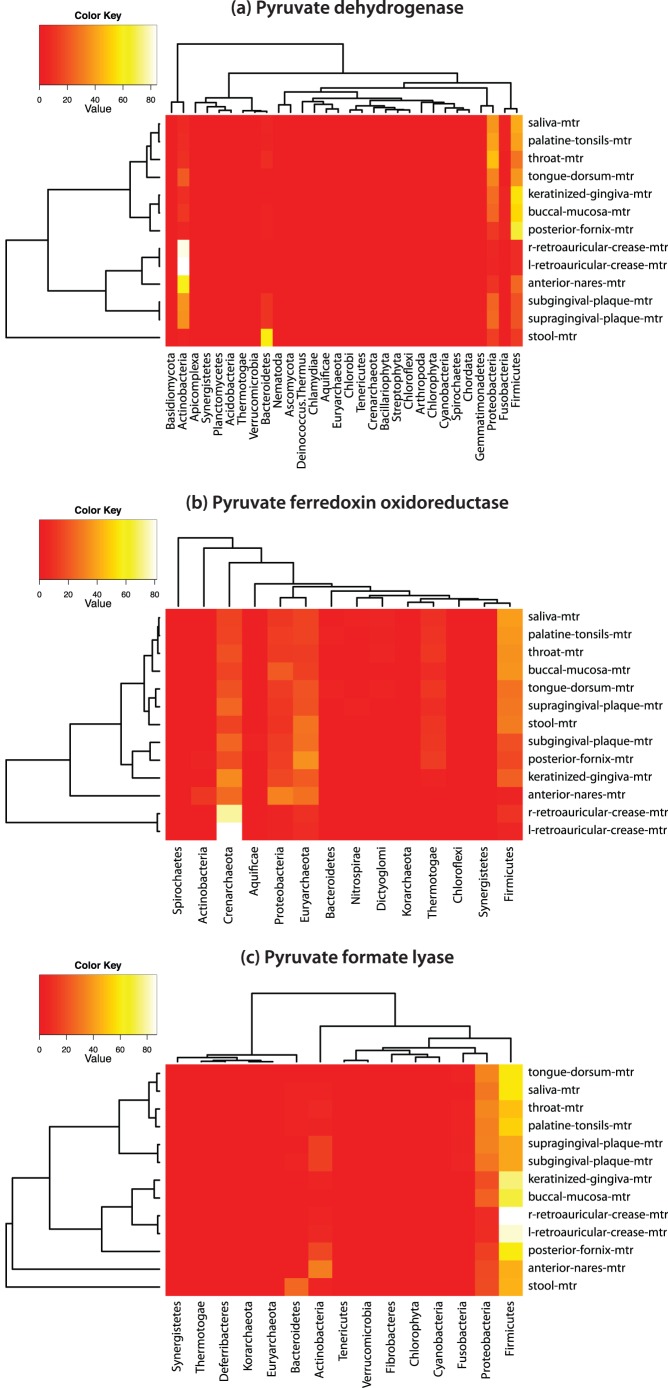
Heatmap plots of three enzymatic markers. Marker abundance is contrasted across phyla (columns) and body habitats (rows) using Morisita-Horn distances in combination with the average linkage clustering method. Colors encode the relative abundance of the selected feature-dataset combination (dark red 0% to white 100%) while the dendograms at the top and left show annotation feature and dataset differences, respectively.

To undertake a comparison of the distribution of these three enzymes across body habitats, analytical functions available through the METAREP Compare page (Figure 1) were employed. To compare the distribution of pyruvate metabolism by taxonomy we filtered pooled datasets from 13 body habitats (n = 493 HUMAnN datasets; 97 donors) for the three pyruvate metabolism enzymes (PDHC, PFOR, PFL) and compared their abundance across taxonomy at the phylum level (see Methods section for details of the filter queries) with multiples distance metrics (Euclidean, Bray-Curtis and Morisita-Horn) to examine the subsequent cluster topologies for consistency. The absolute weighted count matrices (phyla versus body habitats) for each marker enzyme can be found in Table S1. For all of the distance metrics used consistent dendrogram topologies were recovered for all 13 PFOR, 12 PDHC and 10 PFL filtered body habitats (Figure S1). Heatmap plots with dendograms using the Morisita-Horn distance metric are shown in Figure 2.

#### Scenario 1 Results

Results of the PDHC analysis recovered a total of 39 phyla indicating the broad taxonomic distribution of this enzyme complex across prokaryotes and eukaryotes. However, the vast majority of the total abundance (94%) was contributed by five phyla, Actinobacteria (29%), Firmicutes (27%), Proteobacteria (24%), Bacteroidetes (12%) and Fusobacteria (2%). The remaining 6% of the total abundance was contributed by the remaining 34 phyla, with each classification contributing <1% towards the total abundance. The majority of oral habitats, especially the saliva, palatine tonsils, and throat, along with the tongue dorsum, keratinized gingivae, and buccal mucosa clustered together with the posterior fornix to form a cluster driven by high relative abundances of Firmicutes (range 66%–29%) and Proteobacteria (range 12%–47%) (Figure 2a). The anterior nares was positioned mostly closely to the right and left retroauricular crease in a cluster with high abundance of Actinobacteria (range 59%–84%). The subgingival and supragingival plaque formed a separate cluster that was placed most closely to the anterior nares and skin cluster due to variation in the abundance of several phyla, while stool was the most distantly related habitat due to the high abundance of Bacteroidetes (57%).

The analysis of PFL recovered 15 phyla in total, of which 97% of the total abundance was contributed by six phyla, Firmicutes (50%), Proteobacteria (26%), Bacteroidetes (10%), Actinobacteria (7%), Fusobacteria (2%) and Cyanobacteria (2%). The remaining 3% of the total abundance was contributed by the remaining nine phyla, with each classification contributing <1% towards the total abundance. A cluster of oral cavity habitats including palatine tonsils, saliva, throat, tongue dorsum, supragingival and subgingival plaque were recovered in which approximately 50% of the abundance from the body habitat in question was attributed to Firmicutes (range 43%–59%) and approximately one-third to Proteobacteria (range 31%–35%) (Figure 2c). The remaining oral cavity habitats (keratinized gingivae and buccal mucosa) clustered most closely with the right and left retroauricular crease based largely on increased abundance of Firmicutes in these habitats (range 70%–88%). The posterior fornix clustered closest to the skin based in part on a relatively high and similar abundance of Firmicutes (60%) while the anterior nares and stool were the most distantly related body habitats. Although they exhibited similar abundances of Firmicutes (45% anterior nares, 46% stool) they were separated from one another, and the remaining body habitats based on the relatively high abundance of Actinobacteria for anterior nares (32%, highest of all body habitats) and Bacteroidetes in stool (26%, highest of all body habitats) along with variation in other phyla.

In contrast to PDHC and PFL, the analysis of the PFOR recovered a more variable clustering of body habitats within major body regions and very different taxonomic patterns (Figure 2b). In this analysis, 14 phyla were recovered in total of which 95% of the total abundance was contributed by seven phyla, Firmicutes (27%), Euryarchaeota (25%), Crenarchaeota (20%), Proteobacteria (10%), Thermotogae (9%), Actinobacteria (2%) and Dictyoglomi (2%). The remaining 5% of the total abundance was contributed by the remaining seven phyla, with each classification contributing <1% towards the total abundance. The majority of the oral cavity sites, saliva, palatine tonsil, throat, buccal mucosa and supragingival plaque along with stool form one cluster with the highest abundance from Firmicutes and higher abundances of Thermotogae (range 7%–10%) relative to the remaining body habitats. The remaining body habitats revealed the highest abundances in Euryarchaeota and to a lesser extent Crenarchaeota. The left and right retroauricular crease samples were most distantly related to all other body habitats and were dominated by members of the Crenarchaeota (81% and 73%, respectively).

#### Scenario 1 Discussion

The abundances and taxonomic distributions recovered between these three enzymes varied across body habitats; however certain habitats were more likely to be found clustered together and this result was consistent regardless of distance metric used, suggesting closer taxonomic and functional relationships between them. The palatine tonsils and throat which are in close physical proximity within the oral cavity, along with saliva which contacts the entire oral cavity [15], were most consistently clustered together (e.g., have the shortest distances between them) and were most consistently clustered with other habitats from the oral cavity. The subgingival and supragingival plaque which are both biofilms associated with teeth, and the right and left retroauricular crease which are physically disparate from one another, but represent the same skin type [15], were also clustered closest to one another (with the exception of the plaque samples in the PFOR analysis). However their topological positions relative to other habitats from the same body region (oral cavity and anterior nares, respectively) were not consistent regardless of distance metric or clustering algorithm used.

The remaining oral cavity (keratinized gingivae, buccal mucosa, tongue dorsum), stool, anterior nares and posterior fornix exhibited the most variable placement in terms of cluster topology. Taken together, these results suggest that metabolic function can vary across regions of the body and that physical proximity (whether close or separated by relatively greater distances) is not necessarily the most important indicator of taxonomic profile similarity based on the use of functional gene abundance as a biomarker. Instead different habitats can exist within and between body regions that exhibit variable community structure. The exploration of more refined definitions of habitat may be necessary to improve our understanding of microbial biogeography in humans.

In all cases, the taxonomic profiles revealed that the majority of the relative abundance was recovered within a few phyla (5–6). Conversely, more lineages were recovered with low abundance including at least one phylum from the Domain Eukaryota in each example presented. This finding when using functional genes as biomarkers has to our knowledge not been established previously in investigations of the human microbiome. An unusual finding from the examination of the PFOR profiles was the relatively high abundance of Crenarchaeota and Euryarchaeota recovered from the skin habitats as there are few reports of archaea associated with the skin and to our knowledge has not been reported previously using a metabolic marker. The PFOR profile also revealed the presence of lineages less well studied in terms of their associations with humans such as the Thermotogae.

Collectively, these results suggest important new biological insights including: a) the clustering patterns of taxonomic abundance derived from functional genes are not always consistent even when body habitats from similar regions of the body are considered b) the presence of relatively high abundance of Crenarchaeota and Euryarchaeota associated with skin as determined by a metabolic marker (PFOR) and c) that although many lineages (e.g. Thermotogae and the Archaea) may be less prevalent in terms of total abundance within the human microbiome they nonetheless represent an important reservoir of genetic diversity.

### Scenario 2: Sample Variation of Body Habitats and Individuals Across Taxa and Pathways Over Time

#### Scenario 2 Introduction

The nature and extent of variation within and between individuals and body habitats over time is an important topic of study in human microbiome research [30]. Previous studies based on 16S rRNA gene based taxonomic surveys have suggested that microbial community taxonomic profiles were determined largely by body habitat however, interpersonal variability was high within body habitats [31]. More recently, from a metagenomic survey of the human gut microbiome it was suggested that individuals can be grouped based on primarily taxonomic composition and to a lesser extent, functional profiles [7]. Data sets produced by the HMP provide an important opportunity to continue these investigations. Here we hypothesize that although taxonomic and functional composition is expected to vary between samples from different individuals and over time, those samples taken from the same body habitats will be more similar to one another.

#### Scenario 2 METAREP Analytical Methods

The software provides several options to quantify and visualize sample variation that can be used to test this hypothesis. To examine this question and to highlight the hierarchical clustering functionality of METAREP, the variation of taxonomic and pathway composition within and across body habitats and individual donors over two time points was investigated. Data sets from 37 donors (24 males, 13 females) over two sampling time points and 15 body habitats were investigated (84 first and second visit sample pairs, n = 168). A full complement of first and second visits from all 37 individuals across all body habitats was not available. Therefore, certain body sites have a greater contribution of donors with two visits. A breakdown per body site can be found in Table S2.

**Figure 3:**
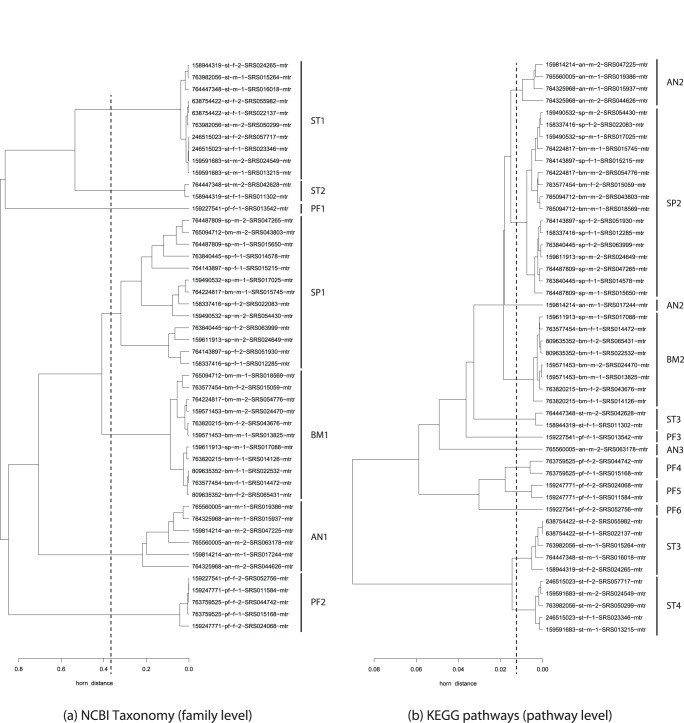
Hierarchical cluster plots of 48 samples taken from 12 females and 12 males at two different time points. Hierarchical clustering analysis of a random subset of human microbiome samples taken from five human body regions clustered by NCBI taxonomy at the family level (a) and by KEGG pathways (b). Clusters were generated by the average linkage clustering method using the Morisita-Horn index to generate a distance matrix (shown on the x-axis). Dataset labels encode the following information [donor ID]-[habitat]-[gender]-[time point]-[sample ID]-[annotation-type]. For example, the dataset label 159814214-an-m-2-SRS047225-mtr encodes a sample from a male donor (ID 159814214) taken from the anterior nares site at time point 2 with sample ID (SRS047225) annotated by the metabolic reconstruction (HUMAnN) pipeline (mtr). The dotted line represents the level at which the tree was cut for analysis. The resulting clusters are labeled as follows: AN (anterior nares), BM (buccal mucosa), SP (supragingival plaque), ST (stool), and PF (posterior fornix).

Datasets were clustered based on taxonomy at the Family level, and function using KEGG pathways. Dendrograms were produced using the Morisita-Horn distance metric in combination with the average linkage clustering algorithm. Initially all samples were clustered in order to visualize the overall patterns produced from these data sets (Figures S2 and S3). For easier visualization of the significant trends determined from the larger data set, a subset of samples (24 first and second visit sample pairs, n = 48) was also clustered (Figure 3). This subset consisted of two sampling time points from 12 males and 12 females taken from five body habitats (Table S2).

#### Scenario 2 Results

The resulting dendrograms (Figure 3, Figures S2 and S3) showed that the majority of samples cluster together based on body habitat using both the taxonomy and functional data sets. The dendrogram topology by taxonomy (Figure 3a, Figure S2) was relatively more consistent in grouping samples from identical or similar body habitats compared to that recovered by function (Figure 3b, Figure S3) in that oral sites were closest to one another followed by samples from the anterior nares, skin and finally vagina and stool. In contrast, the stool, anterior nares and posterior fornix samples produced more variable clustering by function (Figure 3b, Figure S3).

However, exceptions to consistent clustering of samples by body habitat were found within both the taxonomic and functional analyses. For example, the oral cavity sites are dominated by two large clusters, one for supragingival plaque (Figure 3a SP Cluster 1, Figure 3b SP Cluster 2, Figures S2 and S3) and a second for buccal mucosa (Figure 3a BM Cluster 1, Figure 3b BM Cluster 2, Figures S2 and S3). However, in both conditions, there are examples of buccal mucosa samples which cluster with the supragingival plaque and vice versa (Figure 3a SP Cluster 1, Figure 3b SP Cluster 2, Figure 3a BM Cluster 1, Figure 3b BM Cluster 2, Figures S2 and S3) In the dendrogram by function, the anterior nares samples were placed in several locations, including clusters closest to supragingival plaque, (Figure 3b AN Cluster 2), stool (Figure 3b AN Cluster 3) and posterior fornix (Figure 3b AN Cluster 4). Stool samples were broken into three clusters (Figure 3b, ST Cluster 3, ST Cluster 4, ST Cluster 5) with the majority in clusters closest to the anterior nares and posterior fornix (Figure 3b, ST Cluster 4, ST Cluster 5), while two of the samples (Figure 3b ST Cluster 3) were placed closest to anterior nares and buccal mucosa (Figure 3b AN Cluster 3, BM Cluster 2). For the oral cavity body sites with low representation (throat, palatine tonsils, saliva, subgingival plaques, and tongue dorsum) in general it was more difficult to determine the robustness of sample placement within the oral cavity. However, the subgingival plaque samples were always clustered with supragingival plaque in both the taxonomy and functional dendrograms (Figures S2 and S3).

Examination of the temporal component in the dendrograms revealed that for both taxonomy and function in the majority of instances, the first and second time point from a particular individual and body site were not the closest samples to one another. However, these samples were generally found within the same cluster. Data based on the 48 pairwise Morisita-Horn differences strongly supported that differences between first and second time points were significantly lower when compared to all pairwise distances (one sided Wilcoxon rank-sum test p-value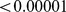). Nevertheless there were notable exceptions. For example, in both the taxonomy and function dendrogram the placement of the posterior fornix sample from the first time point from individual 159227541 (Figure 3a PF Cluster 1, Figure 3b PF Cluster 3) is closest to stool samples (Figure 3a ST Cluster 1, ST Cluster 2, Figure 3b Cluster ST 3), while the second time point from this habitat and individual is closet to other posterior fornix samples (Figure 3a PF Cluster 2, Figure 3b PF Cluster 4, PF Cluster 5). In both the taxonomy and function dendrogram, the first and second time points from the anterior nares from individual 765560005 are not placed closest to one another. In fact, while the first time point is grouped with other anterior nares samples in the function dendrogram, (Figure 3a AN Cluster 1) the second time point is closer to a posterior fornix and stool samples. (Figure 3b Cluster AN 4). In contrast, by taxonomy, although both samples were not closest to one another, they were placed in a cluster of anterior nares samples (Figure 3a AN Cluster 1).

#### Scenario 2 Discussion

In this scenario, taxonomic and functional compositions varied across individuals, body habitats and time although variation within a body habitat was generally less than between habitats as evidenced by the generally consistent clustering of samples by habitat. Exceptions were found which suggest that groups of individuals may exist in which microbiome compositions are more similar to one another and that discrete groups of such individuals could be recovered with taxonomic or functional data. This finding requires more investigation but has important implications concerning the ability to use taxonomic and functional profiles to group individuals. The topology recovered was more variable for function compared to taxonomy. Further, these results suggest that with some notable exceptions, there is generally modest variation in both taxonomy and function in the microbiome within an individual over time. These results could be influenced by technical factors such as some differences in sample coverage, or the relatively greater difficulty of accurately assigning ORFs to pathways compared to taxonomic classifications. Collectively, these results suggest important new biological insights including a) that taxonomy and function are not necessarily coupled, b) that the microbiome can vary across individuals, habitats and time and c) although variation between individuals tends to be higher than between body habitats it may be possible to use taxonomic and functional profiles to group individuals.

### Scenario 3: Detection of Differentially Abundant Taxa and Function between Three Oral Habitats

#### Scenario 3 Introduction

The human oral cavity consists of a variety of surfaces and environments which are colonized by distinct communities of microbial organisms [32]. In the HMP, body habitats sampled from the oral cavity include the buccal mucosa which is the epithelial lining of the cheek and lips, the tongue dorsum, or papillated surface of the tongue, and supragingival plaque which is a biofilm on the tooth surface above the dentogingival junction [10], [15]. Surveys of diversity based on 16S rRNA gene based taxonomic profiles have indicated that over 600 taxa at the species level are found extensively in the human microbiome [33]. Metagenomic data from the HMP now provides an opportunity to extend these analyses beyond 16S rRNA gene based surveys to examinations of taxonomic and functional profiles of distinct habitats from the normal oral cavity. As suggested in previous studies of the oral cavity, and results from Scenarios 1 and 2 in this study, we hypothesize that statistically significant differences in microbial diversity and function are present in HMP metagenomic data from the oral cavity.

#### Scenario 3 METAREP Analytical Methods

To test this hypothesis an analysis was undertaken to determine statistically significant differences in pathways and their associated taxonomic distributions. Specifically, three oral habitats were investigated: 1) buccal mucosa (n = 116), 2) the tongue dorsum, (n = 23) and 3) supragingival plaque (n = 89). These three oral body habitats were selected since they have the greatest representation of WGS data sets in the oral cavity and together constitute more than one fourth of all HMP metabolic reconstruction datasets (Table 1).

**Figure 4:**
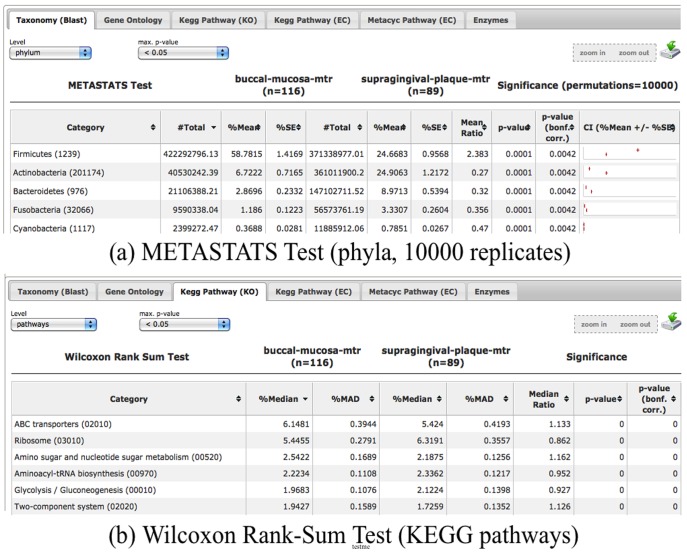
Screenshots of METAREP statistical result panels. List of phyla and pathways that are differentially abundant between the buccal mucosa (n = 116) and supragingival plague (n = 89) habitats. Taxonomic differences reported by Metastats with confidence intervals (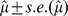) shown in (a), differences in KEGG pathway abundance detected by the Wilcoxon rank-sum test are shown in (b).

The METAREP Compare page (Figure 1) offers two non parametric tests, the Wilcoxon rank-sum test and Metastats, a modified non parametric t-test [34]. Both tests can be used to identify significant differences for a certain annotation attribute between two sample populations. For this scenario, all possible pair wise comparisons between habitats were compared based on taxonomic designations at the phylum level and metabolic functions at the pathway level (Figure 4). A filtering step was applied to this analysis in which ORFs classified as Chordata were removed to eliminate ORFs most likely associated with the human host. This amounted to 0.5% of the three pooled oral habitat datasets. The ability to easily filter using a variety of data set variables demonstrates one of the strengths of METAREP. The statistically significant phyla and pathways determined from both tests were exported as text files(Bonferroni corrected (adj.) p-value<0.05, 10000 Metastats permutations, Table S3 and S4).

#### Scenario 3 Results

Pair wise comparisons of the three oral habitats revealed two significant trends in taxonomic profiles supported by both statistical tests (adj. p-value<0.01). Significant differences were determined in the abundances of the Firmicutes, with this phyla being most abundant in the buccal mucosa, followed by the tongue dorsum, and least abundant in the supragingival plaque habitats. The second significant trend could be seen in the abundance of Actinobacteria. The data supported a decrease in the abundance of Actinobacteria from its highest value in the supragingival plaque, followed by tongue dorsum, to its lowest value in the buccal mucosa (Wilcoxon adj. p-value<0.01). This trend was also supported at the same level of significance by Metastats except for the comparison of buccal mucosa versus tongue dorsum (Metastats adj. p-value = 0.113). In addition to these trends, both tests indicated that Bacteriodetes were significantly less abundant (adj. p-value<0.01) in buccal mucosa when compared to the other habitats. No significant difference in the abundance of Bacterioidetes could be observed between the tongue dorsum and supragingival plaque, however (Wilcoxon adj. p-value = 0.733; Metastats adj. p-value = 0.097).

Pair wise habitat comparisons of pathway attributes revealed differences in their distribution and abundance. In general fewer pathways revealed statistically significant differences in abundance using the Metastats versus the Wilcoxon rank-sum tests, respectively. Supragingival plaque had the highest overall number of enriched pathways (Table 2).

**Table 2:** Number of pathways that are differentially abundant for each statistical test and oral habitat combination.

Wilcoxon	Buccal Mucosa	Tongue Dorsum	Supragingival Plaque	Total (redundant)
buccal	0	39	52	91
tongue	122	0	22	144
plaque	193	123	0	316
**Metastats**	**Buccal Mucosa**	**Tongue Dorsum**	**Supragingival Plaque**	**Total (redundant)**
buccal	0	41	62	103
tongue	54	0	28	82
plaque	133	113	0	246

Rows indicate the habitat in which pathways were significantly overrepresented. Columns indicate the habitat in which pathways were significantly underrepresented. For example, the Wilcoxon rank-sum test found 39 pathways to be enriched in buccal mucosa when compared with tongue dorsum.

Among the key differences supported by both statistical tests were those determined in the abundance of metabolic functions related to antibiotic biosynthesis, pathogenesis and N-glycan biosynthesis (Table 3). For example, the abundances of KEGG pathways related to tetracycline biosynthesis (ko00253), penicillin and cephalosporin biosynthesis (ko00311), and butirosin and neomycin biosynthesis (ko00524) was enriched in buccal mucosa relative to supragingival plaque, conversely biosynthetic pathways related to vancomycin group antibiotics (ko01055), streptomycin biosynthesis (ko00521) and novobiocin were elevated in the supragingival plaque relative to the buccal mucosa. Several differences in pathways related to pathogenesis were also revealed. For example, the pathway describing *Staphylococcus aureus* infection (ko05150) was found to be significantly enriched in the buccal mucosa relative to the supragingival plaque, while epithelial cell signaling in *Helicobacter pylori* infection (ko05120) was elevated in the tongue dorsum relative to supragingival plaque. N-linked protein glycosylation biosynthesis (ko00510) was enriched in supragingival plaque versus buccal mucosa.

**Table 3:** Selection of KEGG pathways found to be differentially abundant in three oral habitats sorted by the ratio of the median abundances.

Ko ID	Pathway	%Median A	%Median B	Median RatioA/B	Wilcoxon adj.p-value	Metastats adj. p-value
**A = buccal mucosa (n = 116) B = supragingival plaque (n = 89)**						
05150	Staphylococcus aureus infection	0.2851	0.1232	2.314	<0.000001	0.0282
00311	Penicillin and cephalosporin biosynthesis	0.063	0.036	1.75	<0.000001	0.0282
00253	Tetracycline biosynthesis	0.2699	0.1888	1.43	<0.000001	0.029
00524	Butirosin and neomycin biosynthesis	0.0647	0.0532	1.216	<0.000001	0.0284
00521	Streptomycin biosynthesis	0.3746	0.4651	0.805	0.000299	0.0284
05120	Epithelial cell signaling in Helicobacter pylori infection	0.1034	0.1338	0.773	<0.000001	>0.05
01055	Biosynthesis of vancomycin group antibiotics	0.0873	0.1276	0.684	<0.000001	0.029
00510	N-Glycan biosynthesis	0.0179	0.0637	0.281	<0.000001	0.0299
**A = buccal mucosa (n = 116) B = tongue dorsum (n = 23)**						
05150	Staphylococcus aureus infection	0.2851	0.1232	2.314	<0.000001	0.028
00311	Penicillin and cephalosporin biosynthesis	0.063	0.036	1.75	<0.000001	0.0282
00253	Tetracycline biosynthesis	0.2699	0.2083	1.296	<0.000001	0.0289
01055	Biosynthesis of vancomycin group antibiotics	0.0873	0.0993	0.879	0.00598	0.0289
00521	Streptomycin biosynthesis	0.3746	0.4651	0.805	0.000299	0.0282
05120	Epithelial cell signaling in Helicobacter pylori infection	0.1034	0.1338	0.773	<0.000001	0.028
00510	N-Glycan biosynthesis	0.0179	0.0362	0.494	<0.000001	0.0299
**A = supragingival plaque (n = 89) B = tongue dorsum (n = 23)**						
00510	N-Glycan biosynthesis	0.0637	0.0362	1.76	<0.000001	0.0299
01055	Biosynthesis of vancomycin group antibiotics	0.1276	0.0993	1.285	<0.000001	0.029
00521	Streptomycin biosynthesis	0.5414	0.4651	1.164	<0.000001	0.0284
05120	Epithelial cell signaling in Helicobacter pylori infection	0.0969	0.1338	0.724	<0.000001	0.0283
05150	Staphylococcus aureus infection	0.0676	0.1232	0.549	0.000004	>0.05

#### Scenario 3 Discussion

Based on the number of pathways which differ in abundance, results from this investigation suggest that the metabolic potential of the buccal mucosa and tongue dorsum are more similar to one another, relative to the supragingival plaque. These differences are also generally consistent with the significant trends determined in the pair wise comparisons of taxonomic profiles in which changes in abundance in Firmicutes and Actinobacteria were greatest between buccal mucosa and tongue dorsum relative to supragingival plaque. These findings may in part be due to the differences in body habitat, for example, the buccal mucosa and tongue dorsum represent microbial communities associated with epithelial cells which are shed over time from soft tissue while the supragingival plaque represents a biofilm adhered to a non-shedding hard surface [35], however this is a result which warrants further investigation.

The differences in pathway distribution determined in this analysis further provide new insights into additional biological drivers related to host-microbial interactions that may play a role in the functional and taxonomic profiles of the microbiome recovered within and between these habitats. First, these results suggest that the ability to synthesize a variety of antibiotics is a function present in the oral microbiome; however this pattern differs between the body habitats examined. The interplay between antibiotic synthesis and resistance in microbial communities has been described as biological warfare where specific antibiotic activity is opposed by resistance determinants and the state of microbial metabolism plays a role in antibiotic susceptibility [36]. Thus, antibiotic production is an important control factor of the colonization and maintenance of microbial community membership, and metabolic function. Next, these results further suggest that even in the oral cavity of normal adult individuals as examined in the HMP, pathways associated with pathogenesis are present in a range of abundances by habitat. These pathways in general share general functions such as surface attachment and invasion of epithelial cells indicating the possible presence of opportunistic pathogens and more generally the presence of microbial mechanisms for colonization of habitats in the human host.

Finally, the glycosylation of proteins is an important, conserved posttranslational modification in eukaryotic organisms including secretory and membrane proteins [37]. Originally described as exclusive to eukaryotes, recent studies have determined their presence in all domains of life [38]. In this study, this pathway revealed a wide taxonomic distribution across eukaryotes (14 phyla) and prokaryotes (19 phyla) however the vast majority (87%) of the abundance of this pathway was determined to be prokaryotic in origin. In bacteria these pathways have been best studied in pathogens where it has been suggested that they are involved in adherence and invasion of eukaryotic cells [39]. The mechanism of N-linked glycosylation is known to occur largely on surface exposed glycoproteins, therefore other functions for these proteins suggested include protection against proteolytic cleavage, enhancement of protein stability or signals for cellular sorting [38]. The presence of differentially abundant pathways of antibiotic production, pathogenesis and N-linked protein glycosylation biosynthesis as determined in this scenario, reveal potentially important control factors of colonization and maintenance of microbial community membership, metabolic function and host interaction in oral habitats. Collectively, these features described here, may in part act as drivers of microbiome community structure and as such contribute to differences in the taxonomy and function between body habitats.

### Software Architecture & Improvements

**Figure 5:**
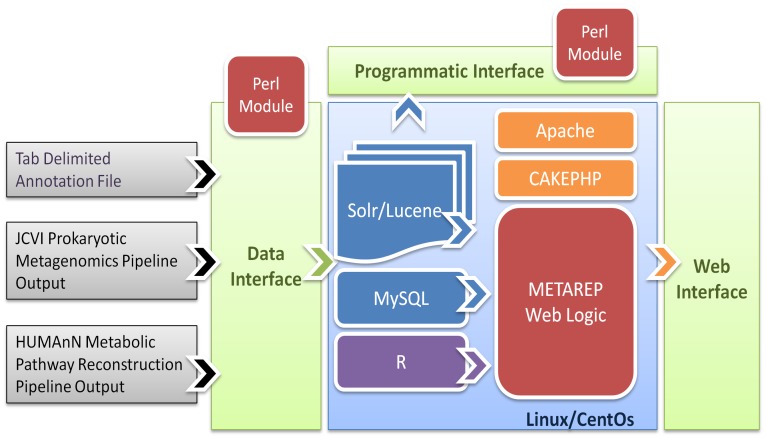
Software architecture overview. The METAREP software integrates several open source tools to import, store and analyze metagenomics annotations. Users can analyze stored data using a variety of web based tools. A subset of the web functionality is available via a programmatic access module which allows data retrieval directly from the MySQL database and Lucene index files.

The software integrates several open source tools and database systems to facilitate the analysis of large volumes of metagenomic annotation data via a web interface [19] (Figure 5). METAREP 1.3.1, adds a programmatic interface to access locally stored data, supports weighted annotations, distributed weighted searches, and functionality to import HUMAnN and JPMAP annotations (see Methods section). New analysis features include browsing, searching and comparing KEGG pathways based on KOs, enhanced clustering via multiple distance matrix options (Morisita-Horn, Jaccard, Bray-Curtis and Euclidean) for generating and visualizing hierarchical clustering, heatmap, and multi-dimensional scaling outputs, and integration of GO slims to summarize GO annotations. The data interface currently supports three annotation formats (Figure 5). The most generic format is a tab-delimited file with rows representing annotation entities (read, transcript, contig, gene, etc.) and columns representing 17 predefined categorical and quantitative annotation attributes (Table 4). The new version, allows users to specify KEGG orthologs and a weight for each annotation to integrate quantitative information. The data backend consists of a MySQL relational database and a non-relational Solr/Lucene full-text search server. The relational database is used to store hierarchical data (NCBI taxonomy, GO, KEGG/MetaCyc pathways, enzyme classification), and dataset and project meta-data information as well as user account information. The Solr/Lucene platform provides fast access to imported annotation data and is used to summarize annotation attribute frequencies. Its faceting functionality is used for unweighted annotations while the statistical component is used for weighted annotations (see Methods section). The R statistical package supports statistical tests and the generation of high resolution PDF plots. The web interface logic is implemented in PHP using the CAKEPHP framework to separate the data access layer from the data representation layer via controller logic (Model View Controller paradigm). Web 2.0 elements are implemented in JavaScript using the jQuery and jQuery UI libraries. Data communication between the PHP controller logic and the Solr/Lucene backend utilizes the light-weight JSON data-interchange format for optimal data transfer. The software includes Perl modules that allow users to automatically download up-to-date versions of the hierarchical data, import annotations, and programmatically access data stored in the Solr/Lucene index files. The latest open source code licensed under the MIT license is available at https://github.com/jcvi/METAREP.

**Table 4:** Column descriptions of the METAREP tab delimited import format.

Column	Field Name	Description	JPMAP	HUMAnN
1	peptide_id	unique entry ID	JCVI_PEP_1234123	ptr:453118
2	library_id	dataset ID	SRS011061	SRS011061
3	com_name	functional description	sugar ABC transporter, periplasmic sugar-binding protein	LGMN; legumain; K01369 legumain [EC:3.4.22.34]
4	com_name_src	functional description source	Uniref100_A23521	ptr:453118
		description assignment		
5	go_id	Gene Ontology ID	GO:0009265	GO:0001509
6	go_src	Gene Ontology source	PF02511	K01369
		assignment		
7	ec_id	Enzyme Commission ID	2.1.1.148	3.4.22.34
8	ec_src	Enzyme Commission source	PRIAM	ptr:453118
9	hmm_id	HMM ID	PF02511	NA
10	blast_tree	NCBI taxonomy ID	246194	9598
11	blast_evalue	BLAST E-Value	1.78E-20	median
12	blast_pid	BLAST percent identity	0.93	median
13	blast_cov	BLAST sequence coverage	0.82	N/A
14	filter	filter tag	repeat	N/A
15	ko_id	KEGG Ortholog ID	N/A	K01369
16	ko_src	KEGG Ortholog Source	N/A	ptr:453118
17	weight	Weight to adjust abundance of assignments	1	43.23

### Scalability

**Figure 6:**
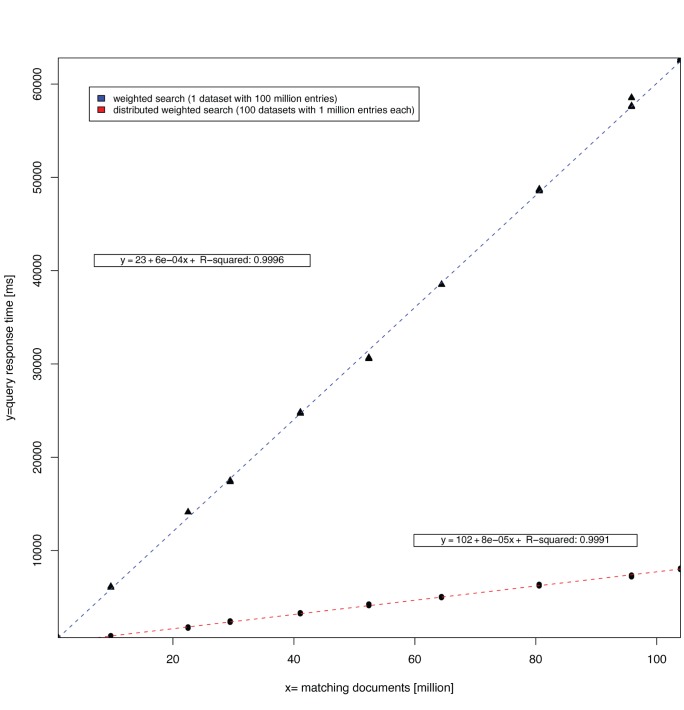
Comparison of query response time for two weighted search approaches. Each data point marks the query response time (y axis) for a query that returned x number of entries (x axis). The blue line indicates the linear fit for the weighted search approach while the red line indicates the linear fit for the distributed weighted search approach. Parameter estimations for the linear regression models are given in the boxes above the fitted lines.

To measure the impact of weighting annotations, the query response time performance was benchmarked using datasets from the buccal mucosa habitat, a collection of 100 samples, each having 1 million entries. Two alternative weighted search approaches were considered, one to search the pooled dataset and one to search the individual datasets in parallel. Thus the overall search volume was kept consistent at 100 million entries. For each search approach, the weighted query response times were recorded for 10 queries that return between 1 and 100 million entries using 10 replicates each. As a baseline, unweighted search times were recorded as well and linear regression analyses were carried out (Figure 6, Table S5). The benchmark was carried out using the hardware as specified in the Methods section. Under the constraints of the hardware and test data, we observed the following. While unweighted searches resulted in response times of less than 52 milliseconds for increasing number of matching entries with slopes not significantly different from 0, the weighted search was proportional to the number of matching entries with response times of up to 62 seconds indicating a time complexity of O(n) (blue and red dotted regression lines, R-squared 0.999). However, weighted results show that given our hardware the distributed search resulted in a 7.9 fold reduction of query response time for any query when compared to the undistributed search (based on the proportion of the two slopes) with a maximum response of 8 seconds.

## Discussion

As sequencing technologies progress, computational methods are constantly being developed or improved to cope with increased throughput and to accommodate changes in the nature of the data. Short read annotation is a special challenge that was accurately addressed by the HUMANnN methodology as part of the HMP project [16]. Given the volume of the data, exploratory analysis and visualization is similarly challenging. In the present study, we show that the current version of our software, has been adapted to handle weighted annotations, and can be used to simultaneously search, compare, cluster, and functionally characterize hundreds of metagenomic samples comprising annotations derived from billions of WGS sequence reads. Other scenarios for integrating weighted annotation schemes include weighting annotations by the number of assembled reads per ORF predicted from assemblies, or quantifying molecules in metatranscriptomics or metaproteomics studies. Our benchmarks indicate that response time increases linearly with an increasing number of weighted entries (we observed an increase of 6.0 seconds per 10 million additional entries). However, for this release of the software we have added functionality to support weighted distributed searches which can significantly improve scalability on multi-core server systems. For our hardware configuration, we observed an increase of 0.8 seconds per 10 million additional entries.

To highlight key functionality of the software, we presented several scenarios designed to analyze the human microbiome. We showed how to analyze a selection of functional markers across taxonomic classifications and body habitats, cluster multiple datasets by functional and taxonomic attributes, and demonstrated how to identify differentially abundant features using statistical tests. We point out that METAREP further possesses many additional features that are not discussed in depth here but provide increased capability to analyze and compare large and complex metagenomic data sets. For example, the Browse Pathways page allows users to visualize enzyme or KEGG ortholog abundances on top of KEGG pathway maps and restrict the results to certain taxa or functions, statistical tests can be applied to a subset of the data, such as enzymatic markers, as well as be used to compare other annotation attributes including MetaCyc, enzyme or the newly implemented GO slim classifications [13]. All analysis features and data including 700 assembly datasets not analyzed in this article are available as a community resource at http://www.jcvi.org/hmp-metarep.

The scenarios presented in this study, while not exhaustive in their scope, have nonetheless highlighted important insights into the human microbiome and generated additional hypotheses for further investigation. Among the key insights we have identified is that first, examination of enzyme profiles by taxonomy provides a mechanism to identify differential abundance of an enzymatic function coupled with the microorganisms contributing the function in question. In this study (Scenario 1), we used this strategy to identify microorganisms that are not abundant across the human microbiome in total such as the Crenarchaeota and Euryarchaeota that nonetheless, revealed an association with skin. These results further suggest that the low abundant taxonomic classifications serve as an important reservoir of genetic diversity in the human microbiome. Next, the variation of taxonomic and functional profiles within body sites is generally less than variation between body sites. Relatively speaking, greater variation occurs across body habitat, individual and time although it may be possible to use taxonomic and functional profiles to group individuals. Further, the link between taxonomic and functional profiles between body habitats is not always coupled. This finding is illustrated particularly in the comparisons of dendrograms based on taxonomic and functional profiles of the PFOR enzyme (Scenario 1) and the examination of metabolic pathways across HMP donor samples (Scenario 2). Finally, examination of the differential abundance of metabolic pathways across three contrasting oral body habitats (Scenario 3) suggests that there are pathways, including many that participate in central intermediary metabolism, which reveal no statistically significant difference between them. This finding implies that there may be common pathways central to the metabolic potential of the oral microbiome. However, there are differences between oral body habitats including antibiotic biosynthesis, pathogenesis and protein glycosylation as identified in this study which may be biological drivers important in the oral microbiome colonization and maintenance, and that contribute to alterations in taxonomic and functional profiles. Collectively, the results of this study indicate the challenge of studying metagenomic data from the human microbiome as it can be influenced by technical artifacts related to sampling, sequencing, and annotation biases, however the application of sophisticated tools for data filtering, analysis and visualization as presented in the METAREP software fundamentally enhance our ability to explore, characterize and interpret these complex data sets.

## Methods

### Ethics Statement

As a part of a multi-institutional collaboration, the Human Microbiome Project human subjects study was reviewed by the Institutional Review Boards at Baylor College of Medicine under IRB Protocol H-22895, the Washington University School of Medicine under protocol number HMP-07-001 (IRB ID # 201105198) and at the J. Craig Venter Institute under IRB Protocol Number 2008-084. All study participants gave their written informed consent before sampling and the study was conducted using the Human Microbiome Project Core Sampling Protocol A. Each IRB has a federalwide assurance and follows the regulations established at 45 CFR Part 46. The study was conducted in accordance with the ethical principles expressed in the Declaration of Helsinki and the requirements of applicable federal regulations.

### Sequence Generation, Preprocessing, and Annotation

DNA was extracted from 108 samples followed by Illumina and 454 sequencing [13]. Low quality regions at the beginning and end of each read were trimmed followed by the removal of sequencing artifacts and human contaminated sequences [13]. Next, preprocessed sequences were assembled by the SOAP de novo assembler into three distinct types of assemblies, Pretty Good Assemblies (HMP Build 1.0 HMASM), Hybrid Assemblies (HMP Build 1.0 HMHASM), and body habitat specific assemblies. ORFs were identified by MetageneMark and annotated using JPMAP [18]. For each predicted peptide, the pipeline ranks and chooses the best evidences obtained by several homology searches including a BLASTP search against UniRef100 [40] and a HMMER3 search against a collection of TIGRFAM and PFAM Hidden Markov models (HMM) [41]. The open source code is available at https://github.com/jcvi/JCVI_HMP_metagenomic_pipeline. The pipeline can be run within Ergatisl [42], a workflow tool that supports compute grid executions. Unassembled reads were annotated by the HUMAnN pipeline [16] which characterizes short reads using an accelerated version of the BLASTX algorithm against a collection of functionally annotated protein databases including KEGG [21] and MetaCyc [22], among others. The software is available at http://huttenhower.sph.harvard.edu/humann.

### METAREP 1.3.1 Installation

To use the software, the METAREP source code and dependent software have to be installed on a Linux based operating system. We recommend users to start with a minimal CentOS 5.5 installation and use the CentOS YUM package installer to install the 3rd party tools. A complete list of YUM packages and detailed information on the installation process can be found on the METAREP WIKI page at https://github.com/jcvi/METAREP/wiki/installation-guide-v-1.3.1. Users can download the METAREP 1.3.1 source via GitHub at https://github.com/jcvi/METAREP/zipball/1.3.1-beta and configure the METAREP instance by editing the application and database configuration files. After successful configuration of the software, users can import annotation data. Import and update scripts can be found under the scripts/perl directory. Example annotations can be found in the data directory.

### Importation of HUMAnN Annotations

We downloaded MBLASTX results against KEGG for 498 datasets from the HMP Data Analysis and Coordination Center (DACC, http://www.hmpdacc.org) and ran HUMAnN v0.8 using its METAREP output format option. The files contain a KEGG gene ID, its median BLAST E-value over all reads, median BLAST percent identity, median read length, and a weight indicating the genes’ relative abundance in the sample. All medians are calculated per gene over all BLAST hits matching it, and weights represent normalized read counts adjusted for individual alignment quality and gene length (comparable to Reads per Kilobase per Million (RPKM) for RNA-seq [43]). We observed a Spearman correlation of 0.94 between the number of reads and sum of the weights for pooled body habitat datasets. For details of the weighting and normalization process mapping reads to genes and orthologous families, see [16]. Example output files can be found in the METAREP installation under the data/humann directory. Next, 498 HUMANnN output files were imported into METAREP using the import script metarep_loader.pl. As part of the HUMANnN indexing process additional KEGG annotation attributes including species name, functional description, KO, EC and GO assignments are fetched from a SQLite database. The database can be created based on downloaded KEGG FTP data (license is required) using the metarep_update_database.pl script.

### Importation of JPMAP Annotations

We downloaded JPMAP annotations for 15 hybrid and 690 pretty good assemblies from the DACC. Next, we loaded the annotations into the HMP METAREP instance using the import script metarep_loader.pl. Example JPMAP output files can be found under the data/jpmap directory.

### Importation of Generic Annotations

To import annotations from other pipelines, data needs to be formatted according to the METAREP tab delimited format specified in Table 4. Examples of tab delimited annotation files can be found under the data/tab directory. Files can be imported using the annotation import script metarep_loader.pl.

### Dynamic Weighting of Annotations

If annotation weights are supplied, absolute frequencies are calculated as the sum of weights of annotation entries that contain a certain annotation attribute. This is accomplished by applying the Solr/Lucene StatsComponent using the weight field as the stats field parameter. Relative frequencies are calculated as the sum of weights of annotation entries that contain a certain feature divided by the sum of all annotation weights. For example, let us assume there are 100 entries in total with weights encoding annotation quality. 80 entries with the KEGG ortholog field (column 15) set to ‘K00849’ (galactokinase). 70 entries out of the 80 have the weight field (column 16) set to ‘8′ while the remaining 10 entries have it set to ‘4′. In addition, there are 20 entries for ‘K00856’ (adenosine kinase), another KEGG ortholog with the weight field set to 20 (high annotation confidence). The relative frequency for feature ‘K00849’ would be 80% if the weights were all equal. Using the new weighting feature the relative frequency is dynamically adjusted to 60%:
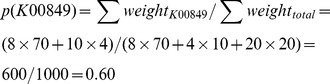



### Scenario Filter Queries

METAREP allows users to filter annotations using the Lucene query language. A query element is specified by the field name to be followed by the value separated by a colon. For example, the query ‘ec_id:1.2.7.1′ retrieves pyruvate synthase entries. Supported search fields are given in column 2 of Table 4. In scenario 1, to filter pooled body habitats for the pyruvate dehydrogenase complex, we searched for ‘ec_id:1.2.4.1 OR ec_id:2.3.1.12 OR ec_id: 1.8.1.4′. Alternatively, the KO attribute can used to filter for the enzyme as well: ‘ko_id:K00161 OR ko_id:K00162 OR ko_id:K00163 OR ko_id:K00627 OR ko_id:K00382’. Filter queries for pyruvate-ferredoxin oxidoreductase and pyruvate-formate lyase were ‘ec_id:1.2.7.1′ and ‘ec_id:2.3.1.54′ respectively. For scenario 3, we filtered the pooled oral habitats for the NCBI taxon Chordata using ‘NOT blast_tree:7711’.

### Hardware

The HMP METAREP instance runs on a single server with two multi-threaded Xeon X7560 2.26GHz processors with a total of 16 cores (32 threads), 256G RAM, and 4 terabyte of disk space.

## Supporting Information

Figure S1 **Impact of distance matrix selection on enzymatic marker based body habitat clustering.** Marker abundance for PDHC (a-c), PFOR (d-f), and PFL (g-i) is contrasted across phyla (columns) and body habitats (rows) using Morisita-Horn, Bray-Curtis and Euclidean distance matrices in combination with the average linkage clustering method.
(PDF)

Figure S2 **Hierarchical cluster plot of 84 first and second visit sample pairs clustered by NCBI taxonomy.** Hierarchical clustering analysis of human microbiome samples with first and second visits (n = 168) taken from 15 human body habitats clustered by NCBI taxonomy at the Family level. Clusters were generated by the average linkage clustering method using the Morisita-Horn index to generate a distance matrix (shown on the x-axis). Dataset labels encode the following information [donor ID]-[habitat]-[gender]-[time point]-[sample ID]-[annotation-type].
(PDF)

Figure S3 **Hierarchical cluster plot of 84 first and second visit sample pairs clustered by KEGG pathways.** Hierarchical clustering analysis of human microbiome samples with first and second visits (n = 168) taken from 15 human body regions clustered by KEGG pathway. Clusters were generated by the average linkage clustering method using the Morisita-Horn index to generate a distance matrix (shown on the x-axis). Dataset labels encode the following information [donor ID]-[habitat]-[gender]-[time point]-[sample ID]-[annotation-type].
(PDF)

Table S1 **Enzymatic marker counts across phyla and body habitats.**
(XLS)

Table S2 **Body habitat and gender statistic for 168 samples with 1st and 2nd visits.**
(XLS)

Table S3 **Differentially abundant phyla (buccal mucosa vs. tongue dorsum).**
(XLS)

Table S4 **Differentially abundant pathways (buccal mucosa vs. tongue dorsum).**
(XLS)

Table S5 **Query response benchmark statistics.**
(XLS)
