# Supplementary material for: A Case Study for Large-Scale Human Microbiome Analysis Using JCVI’s Metagenomics Reports (METAREP)
Source: PLoS One. 2012 Jun 13;7(6):e29044. doi: 10.1371/journal.pone.0029044 (PMC3374610; doi:10.1371/journal.pone.0029044)
Supplement: Figure S1 — Impact of distance matrix selection on enzymatic marker based body habitat clustering. Marker abundance for PDHC (a-c), PFOR (d-f), and PFL (g-i) is contrasted across phyla (columns) and body habitats (rows) using Morisita-Horn, Bray-Curtis and Euclidean distance matrices in combination with the average linkage clustering method. (PDF) [file pone.0029044.s001.pdf]

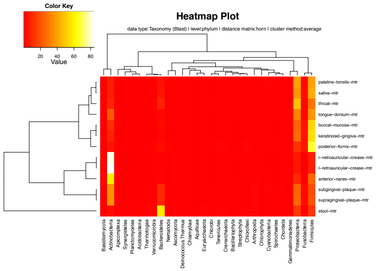

(a) Morisita-Horn PDHC

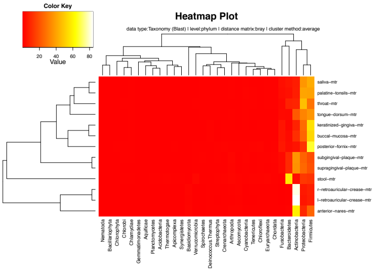

(b) Bray-Curtis PDHC

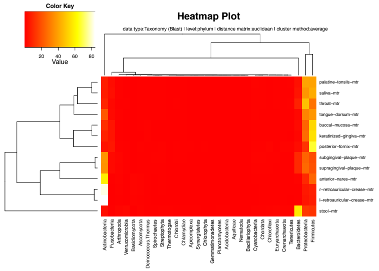

(c) Euclidean PDHC

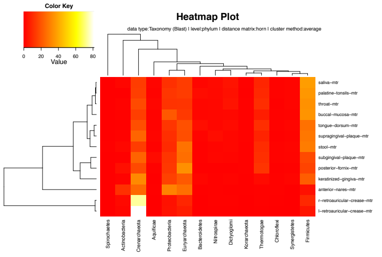

(d) Morisita-Horn PFOR

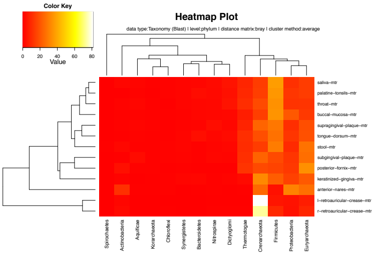

(e) Bray-Curtis PFOR

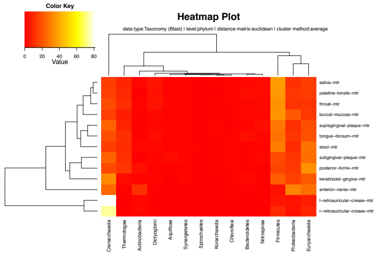

(f) Euclidean PFOR

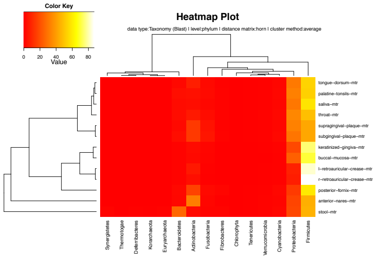

(g) Morisita-Horn PFL

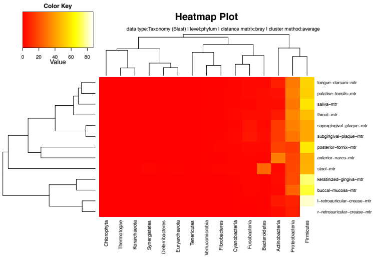

(h) Bray-Curtis PFL

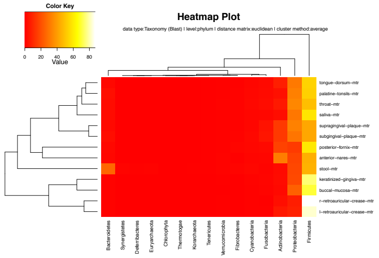

(i) Euclidean PFL
