# Supplementary material for: A Case Study for Large-Scale Human Microbiome Analysis Using JCVI’s Metagenomics Reports (METAREP)
Source: PLoS One. 2012 Jun 13;7(6):e29044. doi: 10.1371/journal.pone.0029044 (PMC3374610; doi:10.1371/journal.pone.0029044)

Hierarchical Clustering Plot

data type:Taxonomy (Blast) | level:family | distance matrix:horn | cluster method:average

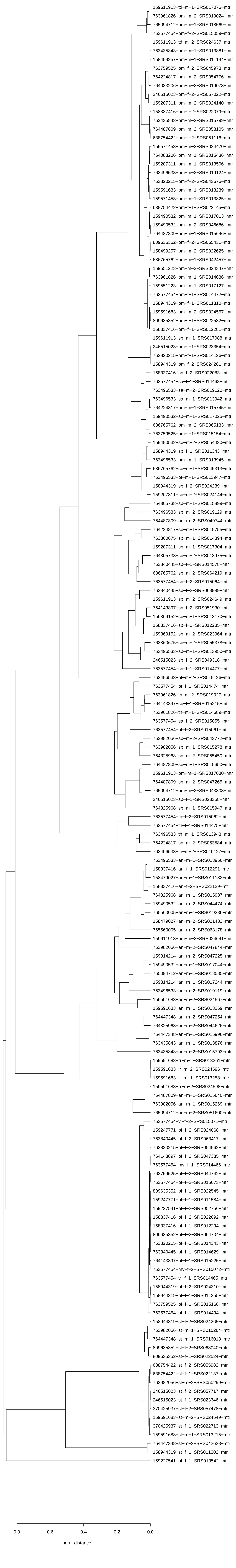

Supplement: Figure S2 — Hierarchical cluster plot of 84 first and second visit sample pairs clustered by NCBI taxonomy. Hierarchical clustering analysis of human microbiome samples with first and second visits (n = 168) taken from 15 human body habitats clustered by NCBI taxonomy at the Family level. Clusters were generated by the average linkage clustering method using the Morisita-Horn index to generate a distance matrix (shown on the x-axis). Dataset labels encode the following information [donor ID]-[habitat]-[gender]-[time point]-[sample ID]-[annotation-type]. (PDF) [file pone.0029044.s002.pdf]
